# Identification of Agents Active against Methicillin-Resistant *Staphylococcus aureus* USA300 from a Clinical Compound Library

**DOI:** 10.3390/pathogens6030044

**Published:** 2017-09-20

**Authors:** Hongxia Niu, Rebecca Yee, Peng Cui, Lili Tian, Shuo Zhang, Wanliang Shi, David Sullivan, Bingdong Zhu, Wenhong Zhang, Ying Zhang

**Affiliations:** 1 Lanzhou Center for Tuberculosis Research and Institute of Pathogenic Biology, School of Basic Medical Sciences, Lanzhou University, Lanzhou 730000, China; niuhongxia1985@163.com (H.N.); bdzhu@lzu.edu.cn (B.Z.); 2 Department of Molecular Microbiology and Immunology, Bloomberg School of Public Health, Johns Hopkins University, Baltimore, MD 21205, USA; ryee2@jhu.edu (R.Y.); shuozhang66@gmail.com (S.Z.); wshi3@jhu.edu (W.S.); dsulliv7@jhmi.edu (D.S.); 3 Key Laboratory of Medical Molecular Virology, Department of Infectious Diseases, Huashan Hospital, Shanghai Medical College, Fudan University, Shanghai 200040, China; keanuc@163.com (P.C.); zhangwenhong@fudan.edu.cn (W.Z.); 4 Beijing Research Institute for Tuberculosis Control, Beijing 100035, China; wishtianlihappy@hotmail.com

**Keywords:** MRSA, antibiotics, repurposed drugs, clinical compound library, MICs

## Abstract

Methicillin-resistant *Staphylococcus aureus* (MRSA) poses a significant threat for effective treatment of several difficult-to-treat infections in humans. To identify potential new treatment options for MRSA infections, we screened a clinical compound library consisting of 1524 compounds using a growth inhibition assay in 96-well plates. We identified 34 agents which are either bacteriostatic or bactericidal against log-phase clinical MRSA strain USA300. Among them, 9 candidates (thonzonium, cetylpyridinium, trilocarban, benzododecinium, bithionol, brilliant green, chlorquinaldol, methylbenzethonium and green violet) are known antiseptics, 11 candidates are known antibiotics currently recommended for the treatment of MRSA. We identified 9 new drug candidates, 5 of which (thiostrepton, carbomycin, spiramycin, clofazimine and chloroxine) are antibiotics used for treating other infections than *S. aureus* infections; 4 of which (quinaldine blue, closantel, dithiazanine iodide and pyrvinium pamoate) are drugs used for treating parasitic diseases or cancer. We ranked these new drug candidates according to their MICs against the MRSA strain USA300. Our findings may have implications for more effective treatment of MRSA infections.

## 1. Introduction

*Staphylococcus aureus* (*S. aureus*) is a significant human pathogen and creates a huge health and economic burden worldwide [1,2]. Methicillin-resistant *S. aureus* (MRSA) is responsible for several difficult-to-treat infections in humans, including skin and soft tissue infections (SSTI), pneumonia, bone and joint infections, bacteremia and endocarditis, and central nervous system (CNS) infections in both healthcare and community settings. Globally, more than 20% *S. aureus* infections are MRSA, and the proportions even exceed 80% in some regions [3,4]. High MRSA proportions pose increased risk for patients and represent a major public health problem, and therefore effective drug treatment for these infections is urgently needed.

In 2011, the Infectious Diseases Society of America (IDSA) published guidelines on the treatment of various types of MRSA infections [5]. The antibiotics recommended by IDSA for MRSA treatment in the clinic include vancomycin, linezolid, daptomycin, tigecycline, telavancin and ceftaroline. However, clinical data for the efficacy of the recommended treatments varies widely [6]. For example, vancomycin treatment just cures 35–57 % of patients with MRSA pneumonia, and it also has low bone penetration and performs poorly in the treatment of *S. aureus* osteomyelitis [7,8], while daptomycin has shown equivalent outcome to vancomycin or even less in the treatment of staphylococcal SSTIs. Due to increasing problem of MRSA, there is recent interest to repurpose clinically used drugs against MRSA [9]. Clinically, MRSA diseases are difficult to cure with current antibiotics partly due to resistance present in both growing and non-growing bacterial populations. According to the Yin-Yang model, bacterial populations consisting of growing and non-growing cells (persisters) can interconvert in vitro and in vivo [10]. Thus, it is possible that targeting both growing bacteria and non-growing persisters may be more effective for treatment of *S. aureus* infections. Previously, we screened a clinical compound library consisting of 1524 compounds for activity against *S. aureus* persisters [11]. Here, as part of our efforts to identify new agents targeting growing bacteria that may be used for the treatment of MRSA infections, we screened the same clinical compound library against log-phase growing *S. aureus* MRSA strain USA300 in this study.

## 2. Results and Discussion

### 2.1. Identification of active hits from the clinical compound library that had good activity against MRSA strain USA300

**Figure 1:**
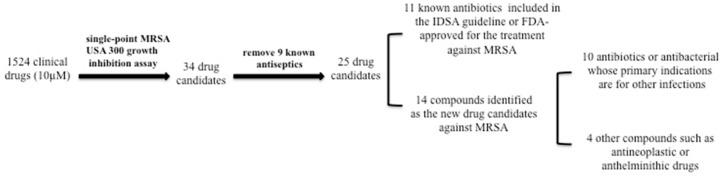
Flow chart of the drug screen procedure to identify drug candidates active against log-phase MRSA USA300 from the clinical compound library.

We screened the clincal compound library for activity against log phase culture of MRSA USA300 using the scheme as described in Methods and Figure 1. Of the 1524 compounds in the clinical compound library, we identified 34 agents that inhibited the bacterial growth completely (Table 1, Table 2 and Table 3). Based on the results of the primary screen, we selected these 34 drug candidates that showed antibacterial activity for rescreens, and the results were found to be reproducible. Among them, 9 drug candidates (thonzonium, cetylpyridinium, trilocarban, benzododecinium, bithionol, brilliant green, chlorquinaldol, methylbenzethonium and gentian violet) are known antiseptics that are used as topical agents (Table 2). Since these antiseptics cannot be used internally for humans, we excluded them from further analysis. However, these antiseptics may be of interest from a mechanistic point of view and may be tested for possible use in disinfecting MRSA contaminated objects or as topical agents for preventing or treating external or surgical wounds.

Among the remaining 25 drug hits, 11 candidates (vancomycin, daptomycin, doxycycline, clindamycin, linezolid, tetracycline, minocycline, meclocycline, chlortetracycline, rifampin, formylrifamycin) are known antibiotics (Table 3), which are currently recommended by IDSA or approved by FDA for the treatment of MRSA [5]. These findings confirm the validity of our screens.

In addition to the known antiseptics and currently recommended antibiotics for MRSA, we identified nine drug candidates (clofazimine, thiostrepton, carbomycin, spiramycin, chloroxine, quinaldine blue, closantel, dithiazanine iodide, pyrvinium pamoate) that had good activity against MRSA USA300 (Table 1). Among the nine drug candidates, five drug candidates (clofazimine, thiostrepton, carbomycin, spiramycin, chloroxine) are antibiotics whose primary indications are for treating other infections than *S. aureus* infections, and four drug candidates (quinaldine blue, closantel, dithiazanine iodide and pyrvinium pamoate) are antimalarial or anthelminthic drugs [12]. The results suggest that MRSA treatment might utilize repurposed drugs previously used for treating other infections or disease conditions. 

### 2.2. Ranking the Activity of the Active Hits by MIC Testing

The most active drug candidates against the MRSA USA300 were rifamycin antibiotics, tetracycline antibiotics, and clindamycin. Doxycycline, tetracycline, minocycline, meclocycline and chlortetracycline are all tetracyclines (TCs), which had relative low MICs and showed high activities against the MRSA strain. In particular, it was worth noting that minocycline and meclocycline seemed to be more active than doxycycline, chlortetracycline and tetracycline against the strain USA300 (Table 3). This finding may be important for choosing the more active tetracyclines such as minocycline and meclocycline over less active doxycycline or tetracycline or chlortetracycline for treating MRSA. Further studies are needed to evaluate if the differences in activity *in vitro* is correlated with clinical outcome. 

It is worth noting that several cell wall inhibitors including nafcillin, cefotiam, cefmenoxime, cefdinir, and moxalactam had good activity against the MRSA strain USA300 (Table 1). It is unclear why these agents had reasonable activity against the MRSA strain. The side chains in these agents may confer some additional antibacterial property as possibly occurs in tosufloxacin [11]. 

To ensure that the hits we identified are also active against other *S. aureus* strains, we tested a few selected drug candidates including clofazimine, closantel, and cefotiam on the *S. aureus* Newman strain in comparison with the MRSA strain USA300 in an MIC test as described in Methods. We found the same MIC for both strains for clofazamine MIC = 5 μM or 2.36 μg/mL. For closantel, a two-fold lower MIC (2.5 μM or 1.66 μg/mL) was found for the Newman strain as compared with USA300 (MIC = 5 μM or 3.31 μg/mL), which is within the allowable variation of MICs for different strains. The only significant difference is with cefotiam where the Newman strain was quite susceptible (MIC= 1.25 μM or 0.65 μg/mL) but the USA300 strain had a four-fold higher MIC (5 μM or 2.62 μg/mL), which may be related to the methicillin resistance in the USA300 strain.

Clofazimine, which was developed as an anti-tuberculosis agent in the 1950s, is currently used in the treatment of leprosy and also used to treat MDR-TB [13,14,15]. It is of interest that we found clofazimine had good activity against MRSA USA300 in vitro. This is consistent with the previous observation that clofazimine has anti-staphylococcal activity and appears to work by disrupting the membrane [16]. On the other hand, as an immunomodulator [17], clofazimine might have the advantage of regulating several aspects of immunity by Wnt signaling pathway for the treatment of chronic or drug-resistant infections in vivo [18]. 

Thiostrepton is a natural macrocyclic thiopeptide antibiotic that inhibits protein synthesis by interfering with the function of elongation factor G (EF-G) [19]. In our previous study, thiostrepton was also found to have activity against *S. aureus* persisters [11].

Closantel was previously shown to have activity against MRSA in vitro [20] and also in *C. elegans* model in vivo [9]. Our result is consistent with these previous findings, indicating the reliability of our assay and results. More importantly, in addition to closantal, we identified other drug candidates (Table 1) that have not been reported as being active against MRSA in previous studies.

Quinaldine blue is an antineoplastic and antimalarial drug, and we found it a high activity against USA300 (MIC = 1.2 μM). Quinaldine (2-methylquinoline) is a heterocyclic quinoline compound that is also used in dye manufacturing, food colorants, pH indicators, and pharmaceuticals [12]. Pyrvinium pamoate is a cyanine dye, a substituted quinoline that has been used to treat pinworm (Enterobius vermicularis) infections. It has antimalarial and anti-cryptosporidium activities [21]. Dithiazanine iodide has broad-spectrum activity against several intestinal parasites and also brain cancer stem cells [22]. Because both of these anthelmintic agents have poor bioavailability and are not well absorbed through the gastrointestinal tract, they may only be used as topical agents for MRSA wound infections. 

While our manuscript was in preparation, we noted a similar study was published by Lau et al. [23]. However, there are several important differences between the two studies. First, we used MRSA strain USA300, while the other study used a different MRSA strain USA100, which may have different susceptibility to antibiotics. Second, we used a larger clinical compound library consisting of 1524 compounds than the other study which used a compound library with 1163 compounds. Third, while the identified known antibiotics share most similarity, the most important difference is the new compounds identified in the two studies are quite different. We identified nine drug candidates (quinaldine blue, thiostrepton, carbomycin, closantel, dithiazanine iodide, clofazimine, pyrvinium pamoate, chloroxine, spiramycin), which do not overlap with the other study that identified only six compounds: ivacaftor, 5-fluorouracil, penfluridol, niclosamide, gemcitabine hydrochloride and floxuridine, as having good activity for MRSA strain USA100 [23]. This is quite surprising. The above differences in bacterial strains, drug libraries, and assay conditions used may account for the very different drug candidates identified by the two different studies.

Since clinical drugs have relatively clear safety and pharmacokinetic profiles in humans and their manufacturing processes have been established, studies examining whether existing clinical drugs that show high activities against MRSA represent a rapid and efficient approach to handle drug-resistant MRSA infections. Future studies are required to evaluate the newly identified drug candidates in drug combinations, especially those drug candidates with low blood concentrations in vitro and in animal models for treatment of MRSA infections before they can be evaluated in patients.

## 3. Materials and Methods 

### 3.1. Bacterial Strain

*S. aureus* strain USA300, a methicillin-resistant strain derived orginally from a clinical isolate from a community-associated MRSA patient, was obtained from BEI Resources (NR-46070). *S. aureus* Newman, is a antibiotics-susceptible strain that was originally isolated from a patient suffering from osteomyelitis [13]. The strains USA300 and Newman were cultured in tryptic soy broth (TSB) for 3 h to log-phase with shaking at 37 °C before it was used for the drug screen or MICs assay as described below.

### 3.2. The Clinical Compound Library

The clinical compound library, also called the Johns Hopkins Clinical Compound Library (JHCCL), consists of 1524 pharmacologically active compounds [24]. The JHCCL compounds were prepared as 10 mM stock solutions and 1 mM working stock in dimethyl sulfoxide (DMSO) [11,24,25] for the drug screen. The first and last columns in each plate were left as blank for controls.

### 3.3. Log-phase Inhibition Screens

MRSA USA300 was revived from frozen glycerol stock on tryptic soy agar (TSA) overnight at 37 °C. Colonies were selected from the agar plate and grown in TSB overnight in a shaker incubator at 37 °C. Then, the bacterial culture was diluted 1:1000 in TSB and grown for 3 h to 1~2 × 10^8^ CFU/mL. Next, the cells were diluted 1:100 in TSB and 100 μL of the bacterial suspension was introduced into each well of the 96-well plates. Then each compound from the clinical compound library in the 1 mM working stock solution (1 μL) was added to the cell suspension to achieve a final drug concentration of 10 μM in the drug screen. The final concentration of DMSO was 1% in each well, which was included as a control. After overnight incubation at 37 °C, optical density (OD600) of each well was determined using a microplate reader. The active hits were confirmed by rescreens using the same procedure as above. 

### 3.4. Minimum Inhibitory Concentration (MIC) Assay

The MIC was determined using the micro-dilution method according to the Clinical and Laboratory Standards Institute (CLSI) guidelines and all the results were repeated three times. MRSA USA300 was revived and grown as the same as for drug screening. Then, the cells were diluted in Mueller-Hinton Broth (MHB) and introduced into 96-well plates. After that, the test antibiotics prepared at 1 mM stock solutions were subjected to two-fold serial dilution in the 96-well plate to make their final concentrations ranging from 10 to 0.3 μM in triplicates. The MIC was defined as the lowest drug concentration (μM) that completely inhibited visible bacterial growth. 

## 4. Conclusions

In summary, we identified 34 agents that have activities against MRSA strain USA300. Among them, 9 candidates are known antiseptics, 11 candidates are known antibiotics used for MRSA treatment, and 9 drug candidates are used for treating other infections (quinaldine blue, thiostrepton, carbomycin, closantel, dithiazanine iodide, clofazimine, pyrvinium pamoate, chloroxine, spiramycin). The 9 drug candidates may be useful for drug combination studies with other antibiotics for more effective treatment of MRSA infections in the future. 

## Figures and Tables

**Table 1:** Drug candidates from the clinical compound library with good activity against log-phase *S. aureus* MRSA USA300.

Drugs	MIC (μM)	MIC (μg/mL)	Original Indication	Cmax * (μg/mL)
Quinaldine Blue	1.2	0.57	Antimalarial	-
Thiostrepton	2.5	4.16	Antibiotic	-
**Nafcillin**	2.5	1.13	Antibiotic	14.34
Carbomycin	5.0	4.20	Antibiotic	-
**Moxalactam**	5.0	2.82	Antibiotic	49
**Cefotiam**	5.0	2.62	Antibiotic	20
Closantel	5.0	3.31	Anthelminthic	-
Dithiazanine Iodide	10.0	5.18	Anthelminthic	-
Clofazimine	10.0	4.73	Antibacterial	0.7–1.0
Pyrvinium Pamoate	10.0	11.51	Anthelminthic	-
**Cefmenoxime**	10.0	5.25	Antibiotic	10800
**Cefdinir**	10.0	3.95	Antibiotic	0.64–1.74
Chloroxine	10.0	2.14	Antibacterial	-
Spiramycin	10.0	8.43	Antibiotic	1000

*Cmax: maximum serum drug concentrations. “-“ indicates “not available”.

**Table 2:** Activity of identified antiseptics against MRSA strain USA300.

Drugs	MIC (μM)	MIC (μg/mL)	Original Indication
**Brilliant green**	1.2	0.58	Antiseptic
**Gentian Violet**	1.2	0.49	Antiseptic
**Bithionol**	5.0	1.78	Antiseptic
**Trilocarban**	10.0	1.56	Antiseptic
**Benzododecinium**	10.0	12.27	Antiseptic
**Chlorquinaldol**	10.0	2.28	Antiseptic
**Methylbenzethonium**	10.0	4.62	Antiseptic
**Thonzonium**	10.0	5.9	Antiseptic
**Cetylpyridinium**	10.0	3.58	Antiseptic

**Table 3:** Activity of identified FDA-approved drugs against MRSA strain USA300.

Drugs	MIC (μM)	MIC (μg/mL)	Original Indication
Minocycline	<0.3	<0.14	Antibiotic
Meclocycline	<0.3	<0.20	Antibacterial
Rifampin	<0.3	<0.24	Antibiotic
Clindamycin	0.3	0.14	Antibiotic
Vancomycin	0.6	0.89	Antibiotic
Formylrifamycin	0.6	0.43	Antibacterial
Doxycycline	0.6	0.30	Antibiotic
Chlortetracycline	1.2	0.62	Antibiotic
Tetracycline	2.5	1.11	Antibiotic
Daptomycin	10.0	16	Antibiotic
Linezolid	10.0	3.47	Antibiotic
